# Is there any difference in organizational commitment between general hospitals and specialized hospitals? Empirical evidence from public hospitals in Beijing, China

**DOI:** 10.1186/s12913-023-10362-5

**Published:** 2023-12-12

**Authors:** Yirui Gao, Junli Zhu, Lujia Hu, Chen Chen

**Affiliations:** Department of Health Policy and Management, School of Public Health, Capital Medical University, Beijing, China

**Keywords:** Public hospital1, General hospital2, Specialized hospital3, Organization commitment difference4, Influencing factors5

## Abstract

**Objective:**

The purpose of the study on the one hand is to see different hospital organization commitment have difference, including the overall score and various dimensions, on the other hand, due to the different hospital type, its function orientation is different, the factors of the doctor organization commitment may also exist differences, so the study of another purpose is to determine for different types of hospital doctor organization commitment the focus and key groups, provide reference for the doctor incentive strategy.

**Methods:**

A total of 292 doctors in four large public hospitals in Beijing were investigated. Physicians’ perceived organizational commitment was investigated using self-made electronic questionnaires. Data were analyzed by factor analysis, descriptive statistics, t-test, ANOVA, and multiple linear regression.

**Results:**

In the large public hospital doctor perception of the hospital commitment status, Specialized hospitals had higher overall commitment behavior scores, it is 3.47 ± 0.86; General hospital commitment behavior scored low at 3.39 ± 0.91. In the regression results, department category, working years, administrative position, and entry mode are the influencing factors of the organizational commitment of doctors in general hospitals, while in specialized hospitals, in addition to whether to hold an administrative position, entry mode, and working hours, the influencing factors also include gender, professional title and overseas learning background.

**Conclusion:**

There are differences in the perceived organizational commitment by doctors in different types of public hospitals, and different factors influencing their organizational commitment.Hospital type directly influences physicians’ organizational commitment and plays a moderating role in influencing other factors. A possible solution is general hospital specialization, encouraging general hospitals to develop the dominant discipline. These findings can help healthcare service hospital executives or government policymakers understand the impact of hospital specialization strategies and develop more efficient medical staff incentive systems.

**Supplementary Information:**

The online version contains supplementary material available at 10.1186/s12913-023-10362-5.

## Introduction

In recent years, with the reduction of medical prices and the development of social security in the forms of subsidies and insurance, more and more people can afford the disease and are willing to see a doctor [1]. To meet people’s daily medical needs, China’s medical resources have been continuously enriched in recent years. Large public hospitals account for most of the total number of hospitals in China. According to the 2021 China Health Statistics Yearbook, by the end of 2020, there were 11,870 large public hospitals in China, including 7,248 general hospitals, accounting for 61.06% of the total hospitals, and 1,818 specialized hospitals, accounting for 15.31% of the total hospitals [1]. However, the carrying capacity of hospitals is too heavy [2], the utilization rate of medical resources is not high [1], and the unreasonable resource allocation is still prominent. How to coordinate and solve the contradiction between the two has become an important topic for us to explore the development mode of different types of hospitals. Due to national policies, economic systems, and other social and environmental factors, the financial support, equipment, and facilities level of specialized hospitals all lag behind that of general hospitals [3]. However, several studies have shown that the fiscal revenue and performance levels of specialized hospitals are higher than those of similar general .

Samiedaluie Studies show that in the United States, as doctors’ workload increases and salaries are generally low, doctors often develop burnout, leading medical students and doctors to give up health care and gradually rejuvenate the health system [3]. In China’s social economy and health system change, there are a lot of negative reports about medical institutions and medical staff, such as doctor-patient conflict, doctor job satisfaction decline, job burnout, resignation, and doctors’ children are unwilling to apply for medical majors, etc. We believe that this may be related to the destruction of the organizational commitments perceived by some doctors, from their lofty career ideals to their helplessness and compromise with reality, and also to the numb acceptance and even internal motivation of some non-public welfare behaviors. But we still believe that this behavior can be improved by specific incentives, such as research by Türk(2014) [4]. Organizational commitment was proposed by the American sociologist Becker in the 1960s to reflect the psychological tacit understanding between the individual and the organization [5–7]. Noval (2016) found that it had a significant impact on job satisfaction, turnover intention, and organizational policies [8]; Herrera (2021) found that the higher the consistency of organizational commitment between the hiring parties, the greater employee satisfaction with the organization, and the more significant organizational civic behavior [9]. Organizational commitment partly reflects that employees work for the benefit of the institution, are eager to stay in it, and the willingness to accept their goals and values [10, 11]. It is often used to study medical staff behavior [12, 13]. To better measure, the hospital commitment perceived by doctors in public hospitals, the researcher, represented by Herriot (1997) [14], believes that the perceptual system of psychological contract exists with both the organization and the individuals, and the employee perception reflects the commitment behavior of the organization to both parties. Combined with the professional characteristics of Chinese doctors, the organizational commitment is divided into four sub-dimensions: transaction, development, relationship, and concept.

Public hospitals are the main body of medical services in China. As social public welfare institutions under the jurisdiction of the government, they are given greater social responsibilities and public expectations. Achieving the goal of public welfare reform depends on doctors who are still in the dominant position of medical services. How guide and to motivate doctors is the key to achieving the goal of reform. Organizational commitment is used to describe the subjective understanding and cognition of employees’ organizational obligations in an organizational exchange relationship. It is regarded as an important perspective and theoretical tool for the study of individual and organizational attitude and behavior [15] and has received wide attention from the management community. Throughout the research [16–18] on medical staff’s perceived organizational commitment, most of the research only focused on a specific group of doctors or nurses, but they less conducted a comparative analysis of doctors’ perceived organizational commitment in different types of hospitals. A clear understanding of the doctors ‘organizational commitments with different characteristics can more effectively identify the focus and key groups to maintain doctors’ organizational commitments, and carry out more targeted employee incentives. The main function orientation of the public large hospitals is to provide high-level scientific research and teaching services in several regions, It also accepts referrals from the secondary and lower hospitals. Its coverage covers a wider range of services and more numbers and types of doctors so that its sample data is more representative and feasible. This study attempts to explore the perceived status and characteristics of hospital commitment by different categories of large public hospital doctors in organizational commitment, analyzing the factors affecting their organizational commitment and providing a reference for doctors’ incentives.

## Data and methods

The cross-sectional survey took place in Beijing, China, from September to December 2019. The survey uses stratified random sampling to select 4 large public hospitals, including 2 large general hospitals (Beijing Tongren Hospital, Beijing Shijitan Hospital), 2 large specialized hospitals (Beijing Anding Hospital, Beijing Hospital of Traditional Chinese Medicine) hospital) composition. Serving physicians in randomly selected hospitals were surveyed as respondents. All respondents were provided with printed information about the study and obtained written informed consent before participating in the survey. This study has been approved by the Ethics Committee of Capital Medical University. Moral issues were considered when designing the project. All of the participants provided a written informed consent form. The participants’ information was anonymous. Participants were randomly selected and asked to fill out questionnaires based on their own actual experiences. The investigators then randomly distributed questionnaires to physicians in each department who met the inclusion and exclusion criteria. A total of 321 physicians were assessed. After deleting respondents with less than one year of work, those in management positions, and questionnaires with missing values, the final sample consisted of 292 physicians.

The questionnaires included “General Information Status” and “Hospital Organizational Commitment Behavior Scale”. General information status includes personal characteristics (age, gender, marriage, education, overseas study experience) and occupational characteristics (hospital category, subject category, professional title, entry method, administrative position) and other items.The questionnaire has been uploaded as supplementary material.According to the features of Chinese doctors, Freese et al. (2008) studied the dimensions of Chinese doctors’ psychological contract questionnaire for the characteristics of Chinese doctors [19]. Based on this study, and met Herrera and De’s psychological contract scale design a set of criteria: evaluation structure must be based on theory; evaluation must meet the content and structure validity (based on empirical research); evaluation must meet the psychological contract measurement characteristics and the degree of a sample. Differences in industry characteristics and occupation types will make the content, structure, and focus of employees’ psychological contracts different [8]. The researchers designed the questionnaire dimension, including transaction, relationship, development, and concept, with a total of 17 items. See Table 1 for the specific entries.The definition and specific content are shown in Table 2. It used a 5-point Likert scale ranging from 1 (never promise) to 5 (very clear promise). All dimensions were greater than 0.7, and the P of the validity correlation coefficient was less than 0.05, indicating the internal reliability and validity of the questionnaire are good.

**Table 1:** Specific items of the hospital organizational commitment

Related dimensions	Specific items
Concept organization commitment	The work provided by the hospital is challenging
	The hospital gives you the autonomy in your work
	Hospitals will take their doctors’ opinions into full account when making major decisions
Development Organization Commitment	The hospital allows you to use your skills and expertise
	The hospital provides you with opportunities for professional learning and technical training
	The hospital provides you with career promotion space and development opportunities
Trading organization commitment	The hospital will pay your salary and bonus according to your work performance
	The hospital provides you with stable job security
	The hospital provides you with a fair and reasonable treatment
	The hospital provides you with superior benefits (such as insurance and vacation)
	The hospital provides you with the conditions and resources needed to carry out your work
Relationship Organization Commitment	The relationship between the superior and lower levels in the hospital is harmonious and friendly
	The hospital maintains a harmonious relationship between colleagues
	The hospital has great respect for its doctors
	The hospital provided a collaborative working atmosphere
	Hospitals care for your personal development and personal life conditions
	The hospital recognizes your contribution and performance

**Table 2:** Definition and content of the hospital organizational commitment

Dimension	Definition	Content
Trading organization commitment	This is the commitments of the hospital to abide by the rules and regulations in the work on time and exchange the economic and material equivalent. It emphasizes the financial benefits and material conditions that the hospital commitment to provide for doctors.	The hospital promises to provide reasonable salary, fair treatment, appropriate working environment and occupational safety protection, etc.
Development Organization Commitment	This is the hospital’s commitment to provide support for doctors’ personal development and capacity improvement. It emphasizes that hospitals attach importance to the personal occupation of doctors and the development prospect of hospital undertakings	The hospital promises to provide good training opportunities, fair promotion channels, and it pay attention to the personal career development of doctors, etc.
Relationship Organization Commitment	This refers to the hospital’s commitment to provide a harmonious interpersonal environment and organizational care to doctors. It emphasizes the long-term emotional maintenance bond between doctors and hospitals.	The hospital promises to provide a good way to communicate with managers and promote good cooperation among doctors.
Concept organization commitment	This is the concrete action of hospitals to attract doctors to realize their organizational concept with their own value.It emphasizes encouraging doctors to maintain their organizational values and beliefs beyond material things.	The hospital creates an organizational atmosphere to strengthens the staff’s organizational culture research.

The research’s dependent variable was the physician “hospital commitment Behaviour Scale” score from two types of hospitals. However, the independent variables included 10 variables, of which two categories were sociodemographic characteristics and job characteristics. Doctors with high empirical education to Anderson et al. (2021) have significantly increased scores on hospital commitment [20]; Gider et al. (2019) pointed out that doctors with different professional titles perceived noticeable differences in hospital concept commitment and relationship commitment behavior [21], and Kim et al. (2017) proved that marriage, gender and subject category did not affect doctors’ subjective understanding and cognition of mutual obligations in a hospital exchange relationship [22]. Finally, we determined these 10 representative variables after extensive reading of relevant literature combined with the actual situation in China and set various parameters at the same time.

Descriptive statistics of the study variables are reported using frequency and percentage. The statistical analysis takes three steps to study the impact of different hospital types, sociodemographic characteristics, and work characteristics on hospital organizational commitment. The first step is an exploratory factor analysis of the sample tissue commitment score to determine whether the factor component is the same as the initial questionnaire setting. The second step is a univariate analysis of essential characteristics and hospital commitment scores using an independent sample t-test, ANOVA. The third step is the multiple-wise linear regression. Hospital organizations promise to group hospitals according to the type of hospital, whether a public general hospital or a public specialized hospital. Each group is classified by the total dimension and each sub-dimension of the “Hospital Organization Commitment Scale”. Univariate analysis is a preliminary exploration of the association between independent and dependent variables, and binary regression analysis aims to exclude the influence of other confounding factors further and ultimately determine the correlation between independent and dependent variables. All statistical analysis is implemented through SPSS 26.0.

## Results

In total, we surveyed 292 doctors from four large public hospitals in Beijing. 210 Of the 292 doctors are from public general hospitals (Beijing Tongren Hospital, Beijing Shijitan Hospital). Others are from public specialized hospitals(Beijing Anding Hospital, Beijing Hospital of Traditional Chinese Medicine). Doctors who hold administrative positions, enter the hospital by campus recruitment, and work for less than 10 years think that hospital organizational commitment has higher scores. The results of univariate analysis and regression show that department category, working years, whether to hold an administrative position, and entry mode are the influencing factors of the organizational commitment of doctors in general hospitals, while in specialized hospitals, in addition to the administrative position, entry mode and working hours, the influencing factors also include gender, professional title and overseas learning background.

### Exploratory factor analysis

The KMO of the total commitment behavior scale of physicians in public hospitals was 0.941, and the Barlett sphericity test was *P* < 0.05. Principal component analysis was used for the factor analysis, resulting in four factors with a cumulative explained variance of 77.858%. The factor composition is the same as the reference questionnaire, named as development commitment, transaction commitment, relationship commitment, and concept commitment (Table 3). Among them, the relationship commitment behavior of public hospitals is at the middle level, the concept commitment behavior is at the lower middle level, and the transaction commitment behavior is all above the middle level.

**Table 3:** Differences in the organizational commitments (x ± s)

Dimension	Number of entries	Maximum	Minimun	Total average score		
				General hospital	Specialized Hospital	Total
Relationship commitment	6	5	1	3.43 ± 0.95	3.38 ± 0.73	3.42 ± 0.82
Development commitment	5	5	1	3.42 ± 0.97	3.14 ± 0.67	3.49 ± 0.90
Transaction commitment	3	5	1	3.52 ± 1.00	3.61 ± 0.72	3.55 ± 0.93
Concept commitment	3	5	1	3.15 ± 1.10	3.67 ± 0.72	3.14 ± 1.04
Total	17	5	1	3.39 ± 0.91	3.47 ± 0.86	3.41 ± 0.82

### Sociodemographic characteristics and occupational demographic characteristics

The basic information of the 292 surveyed physicians surveyed is shown in Tables 4 and 5. Overall, 292 physicians returned their questionnaire (100%), and their perceived score for organizational commitment behavior was 3.41 ± 0.82. The majority of the doctors surveyed were women (63.4%), aged between 25 and 45 (82.9%), with a master’s degree or above (69.9%), and unmarried (78.8%). Of these, 252 doctors had no overseas study experience (86.3%). The majority of doctors surveyed were from general hospitals (71.1%), with 149 doctors from non-key disciplines (51.0%) and 143 doctors who did not hold administrative positions (49.0%). Most doctors entered the hospital through fresh entry (83.6%), and the title of attending physician or above (68.8%).

**Table 4:** Univariate analysis: organizational commitment difference score (x ± s): ——Different individual social characteristics

Category	Number of people (n%)	Hospital commitment score					t/f
		Relationship commitment dimension	Development commitment dimension	Transaction commitment dimension	Concept commitment dimension	Total commitment dimension	
Sex							
Man	107(36.6)	3.39 ± 0.95	3.46 ± 0.96	3.50 ± 0.96	3.22 ± 1.07	3.40 ± 0.87	-0.202
Woman	185(63.4)	3.43 ± 0.86	3.51 ± 0.87	3.60 ± 0.91	3.10 ± 1.02	3.42 ± 0.80	
Age							
25 ~ 35	129(44.2)	3.42 ± 0.89	3.54 ± 0.92	3.55 ± 0.94	3.10 ± 1.06	3.42 ± 0.85	0.705
36 ~ 45	113(38.7)	3.36 ± 0.89	3.39 ± 0.90	3.58 ± 0.88	3.22 ± 1.00	3.38 ± 0.80	
≥ 46	50(17.1)	3.52 ± 0.93	3.28 ± 0.86	3.45 ± 0.99	3.12 ± 1.09	3.46 ± 0.81	
Marital status							
Unmarried	230(78.8)	3.41 ± 0.89	3.48 ± 0.90	3.52 ± 0.92	3.13 ± 1.02	3.40 ± 0.79	-0.609
Married	62(21.2)	3.44 ± 0.91	3.54 ± 0.91	3.63 ± 0.95	3.19 ± 1.08	3.46 ± 0.86	
Educational background							
Doctoral candidate	86(29.5)	3.34 ± 0.91	3.48 ± 0.89	3.46 ± 0.95	2.95 ± 1.03	3.33 ± 0.77	1.497^**^
Master Degree Candidate	118(40.4)	3.43 ± 0.91	3.54 ± 0.91	3.43 ± 0.91	3.20 ± 1.05	3.45 ± 0.86	
Bachelor and below	88(30.1)	3.46 ± 0.86	3.45 ± 0.87	3.54 ± 0.90	3.26 ± 1.01	3.54 ± 0.82	
Overseas study experience							
Yes	40(13.7)	3.26 ± 1.01	3.35 ± 0.98	3.48 ± 1.03	3.10 ± 1.12	3.30 ± 0.91	-0.965
No	252(86.3)	3.44 ± 0.87	3.51 ± 0.89	3.56 ± 0.91	3.15 ± 1.04	3.41 ± 0.82	

*0.10; **0.05; ***0.01

**Table 5:** Single factor analysis: organizational commitment difference score (x ± s) ——Different individual occupational characteristics

Category	Number of people (n%)	Hospital commitment score					t/f
		Relationship commitment dimension	Development commitment dimension	Transaction commitment dimension	Concept commitment dimension	Total commitment dimension	
Department category							
Key disciplines	149(51.0)	3.47 ± 0.87	3.54 ± 0.86	3.64 ± 0.91	3.28 ± 1.00	3.49 ± 0.79	1.632
Non-key disciplines	143(49.0)	3.35 ± 0.91	3.44 ± 0.94	3.44 ± 0.94	3.01 ± 0.94	3.33 ± 0.85	
Whether to hold an administrative position							
Yes	134(45.9)	3.95 ± 0.86	3.89 ± 0.92	3.79 ± 1.21	3.33 ± 1.22	3.80 ± 0.89	1.722^*^
No	158(54.1)	3.39 ± 0.89	3.47 ± 0.90	3.53 ± 0.91	3.14 ± 1.03	3.39 ± 0.82	
Entry method							
Campus recruitment	244(83.6)	3.46 ± 0.86	3.54 ± 0.87	3.64 ± 0.84	3.20 ± 1.03	3.47 ± 0.78	-2.444^**^
Transfer of other institutions	48(10.6)	3.10 ± 1.08	3.12 ± 1.06	3.11 ± 1.13	2.84 ± 1.12	3.06 ± 1.01	
Positional titles							
Associate Chief physician or above	102(34.9)	3.23 ± 1.00	3.28 ± 0.92	3.43 ± 1.02	3.14 ± 1.03	3.26 ± 0.90	-0.989
Physician	99(33.9)	3.33 ± 0.86	3.38 ± 0.91	3.53 ± 0.85	3.16 ± 0.98	3.35 ± 0.77	
Physician and below	91(31.2)	3.47 ± 0.93	3.60 ± 0.93	3.61 ± 0.94	3.14 ± 1.11	3.47 ± 0.88	
Working hours (Years)							
≤ 10	163(55.8)	3.43 ± 0.87	3.50 ± 0.92	3.59 ± 0.90	3.15 ± 1.03	3.43 ± 0.83	1.899^*^
>10	129(44.2)	3.39 ± 0.89	3.38 ± 0.90	3.50 ± 0.93	3.04 ± 1.039	3.38 ± 0.82	

*0.10; **0.05; ***0.01

### Results of the univariate analysis

The univariate analysis affecting the hospital organizational commitment scores is shown in Tables 4 and 5. In terms of sociodemographic characteristics, the univariate analysis showed that this was considered significant among doctors with different educational backgrounds (*P* < 0.05), and ANOVA found that doctoral candidates had higher scores than those with other degrees (*P* < 0.05). In terms of the occupational demographic characteristics, there was a statistically significantly different in the score of hospital commitment behavior between doctors who held administrative positions and those who did not hold administrative positions (*P* < 0.1). There was a significant difference in the scores of doctors in different entry modes (*P* < 0.05). The analysis of variance found that the scores of doctors transferred from other institutions were lower than those in other entry modes. The scores of doctors with different working years were statistically significant (*p* < 0.1), and ANOVA found that the perceived organizational commitment behaviors were higher for doctors with working years < 10 years.

### Results of the multiple linear regression

Table 6 introduces the multiple linear regression results of the influence of various organizational commitment behaviors of different public hospital types. The multiple linear regression equations (*p* < 0.1) fitted by each model can be considered statistically significant. The regression results of all physicians indicated that whether to hold administrative positions and entry methods were the main influencing factors of doctors’ perceived organizational commitment. In addition, working hours influence development commitment scores(*P* < 0.10).

**Table 6:** Multiple linear regression: Analysis of factors affecting differences in overall organizational commitment

	Total commitment		Development commitment		Relationship commitment		Transaction commitment		Concept commitment	
	General hospital (*N* = 210)	Special hospital (*N* = 82)	General hospital (*N* = 210)	Special hospital (*N* = 82)	General hospital (*N* = 210)	Special hospital (*N* = 82)	General hospital (*N* = 210)	Special hospital (*N* = 82)	General hospital (*N* = 210)	Special hospital (*N* = 82)
Variable	Β/t	Β/t	Β/t	Β/t	Β/t	Β/t	Β/t	Β/t	Β/t	Β/t
Sex	-0.008 (-0.234)	0.316 (20.451)^**^	-0.129 (-0.922)	0.309 (20.009)^**^	-0.140 (-10.022)	0.411 (20.453)^**^	-0.050 (-0.350)	0.148 (0.838)	-0.237 (-10.498)	0.327 (20.157)^**^
Age	0.066 (0.532)	-0.043 (-0.388)	-0.034 (-0.203)	0.191 (10.218)	0.145 (0.889)	-0.021 (-0.147)	0.089 (0.524)	0.039 (0.214)	0.192 (10.018)	-0.356 (-10.225)
Marriage	0.045 (0.146)	-0.036 (-0.249)	-0.098 (-0.530)	-0.071 (-0.384)	-0.022 (-0.124)	-0.012 (-0.064)	-0.084 (-0.450)	-0.006 (-0.027)	-0.024 (-0.116)	-0.077 (-0.358)
Department category	-0.151 (-10.564)	-0.058 (-0.446)	-0.157 (-10.129)	0.096 (0.607)	-0.173 (-10.275)	-0.068 (-0.402)	-0.304 (-20.161)^**^	0.119 (-0.649)	-0.323 (-20.051)^**^	-0.301 (-10.493)
Educational background	-0.041 (-0.246)	-0.115 (-10.230)	0.046 (0.462)	-0.104 (-0.793)	0.000 (0.004)	-0.168 (-10.385)	0.075 (0.740)	-0.027 (-0.180)	-0.184 (-10.626)	0.223 (10.470)
Positional titles	0.035 (0.547)	-0.104 (-0.947)	0.225 (10.586)	-0.150 (-10.009)	0.190 (10.373)	-0.146 (-10.018)	4.892 (22.280)^***^	-0.112 (-0.656)	0.190 (10.187)	0.208 (-10.450)
Whether to hold an administrative position	-0.407 (-10.880)^*^	-0.557 (-20.200)^**^	-0.732 (-20.465)^**^	-0.359 (-10.370)	-0.720 (-20.482)^**^	-0.699 (-20.545)^**^	-0.454 (-10.513)	-0.534 (-10.768)^*^	-0.242 (-0.721)	-0.675 (-20.442)^**^
Working hours (years)	-0.071 (-0.469)	-0.087 (-0.156)	-0.340 (-20.430)^**^	-0.355 (-30.025)^**^	-0.101 (-0.450)	0.018 (0.026)	0.017 (0.073)	-0.011 (-0.087)	-0.026 (-0.099)	0.161 (0.192)
Entry method	0.008 (-20.016)^**^	-0.0632 (0.562)^**^	0.145 (-20.215)^**^	-0.401 (-20.111)^**^	0.039 (-20.376)^**^	-0.034 (20.782)^**^	0.053 (-30.900)^***^	-0.518 (60.938)	0.051 (-10.762)^*^	0.371 (10.907)^*^
Overseas study experience	-0.465 (10.209)	0.409 (-20.421)^**^	-0.282 (0.833)	-0.817 (20.562)^**^	-0.295 (10.609)	0.577 (-10.817)^*^	-0.501 (0.443)	40.203 (0.838)	-0.253 (10.271)	0.468 (-0.860)
f	10.909^**^	20.178^**^	10.712^*^	20.123^**^	10.880^**^	20.051^**^	20.393^**^	20.166^**^	20.038^**^	20.320^**^

*0.10; **0.05; ***0.01

Multiple linear regression analysis is used to explore the influence of organizational commitment in both groups. The two regression results show that the department category is the main factor affecting the transaction commitment and concept commitment in public general hospitals. Overseas study experience is the main influent factor for doctors in public specialized hospitals to the overall organizational commitment, development commitment, and relationship commitment. Gender is the influent factor in the concept commitment, development commitment, and relationship commitment in public specialized hospitals (*p* < 0.1).

## Discussion

This research discussed the differences between different large public hospitals based on the organizational commitment behavior of four large public hospitals in Beijing and analyzed the influencing factors to provide a basis for different types of hospitals to formulate a more precise talent incentive system.

This research find that the impact pathway of hospital type on doctors’ perceived organizational commitment was complex. On the one hand, the total organizational commitments in specialized hospitals were higher, especially for transaction commitments and conceptual commitments. The reason may be that due to the difficulty of seeing a doctor in general hospitals in China in recent years, policymakers have guided patients to divert them, which has brought rapid development to a large number of specialized hospitals. Cui [23] et al. (2021) pointed out that the operational efficiency of public specialized hospitals in China is higher than that of public general hospitals. Their advantages are reflected in indicators such as average hospitalization days, drug ratio, daily income per bed, and capita income. Due to the concentration of disciplines, the organizational culture construction of specialized hospitals is more unified than that of general hospitals. Rozier points out that it is more urgent for specialized hospital doctors to integrate into the organizational culture, and doctors have more autonomy in their work [24]. On the other hand, there were differences in perceived organizational commitment factors between the two hospitals. In the results of multiple regression, administrative position and entry mode are the influencing factors of doctors’ commitment to organizations in the general hospital; However, in specialized hospitals, in addition to whether to hold an administrative position, entry method, the influencing factors also include gender and overseas study background. Thus, it appears that hospital type directly affects physicians’ perceived organizational commitment and plays a regulatory role in influencing other factors. Indeed, although most researches show that specialized hospitals operate more efficiently than general hospitals and have the added convenience of personnel management, the number of public hospitals is much larger than specialized hospitals [25, 26]. Therefore, McCarthy et al. (2008) suggest that a possible solution is general hospital specialization, encouraging general hospitals to develop the dominant discipline [27]. China is exploring the mode of “specialty” in modern general hospitals, which leads to apparent differences in the development and training resources given to various clinical disciplines in general hospitals. Due to the variety of clinical disciplines and the more complex personnel management system in general hospitals, there are obvious differences in the organizational commitments made by general hospitals to different doctors in different fields [23]. These findings can help healthcare service hospital executives or government policymakers understand the impact of hospital specialization strategies and develop more efficient medical staff incentive systems.

Another finding of this study is that the department category only affects the transaction commitment, and concept commitment of general hospitals, and not specialty hospitals. The reason may be that the department type of specialized hospitals is more single than that of general hospitals. Research from the UK NHS showed that hospital specialization contributes to improving hospital and patient satisfaction efficiency, but it cannot replace the convenience brought by comprehensive facilities. Unsurprisingly, China has already moved in that direction by creating a number of the policy document [12, 23]. Under the guidance of policies, general hospitals should strengthen the development of the key disciplines, and the treatment and development opportunities of doctors in their key disciplines and non-key disciplines are obviously different. Specialized hospitals are required to develop comprehensively and gradually strengthen the support for the construction of non-key disciplines. We also found that physicians in key disciplines have higher transaction commitment and concept commitment. The reason may be that the rights and interests of doctors in key disciplines, such as work remuneration and promotion paths, are better than those of doctors in non-key disciplines. Moreover, the hospital pays more attention to the assessment of personnel in key disciplines and the construction of department culture, which makes the working atmosphere in the department better [27]. Indeed, several past perceived organizational commitment types of research of physicians in public hospitals have drawn similar conclusions [28–30].In a cross-sectional survey, Chinese scholars Liu et al. (2019) note that the hospital committed to improving medical staff in non-key disciplines helps to improve the overall operating efficiency and staff satisfaction of hospitals [31]. This finding indicates that hospitals should value the organizational commitment of non-key personnel, such as improving their compensation and personnel management commitments, while changing the idea that non-key discipline doctors are not valued. Let them feel the care of the organization, and thus improve the perception of organizational commitment.

This research found that the hospital transaction commitment was somewhat different when it was perceived by general hospital physicians with different professional titles, and the professional title grade was positively correlated with the score of transaction commitments. However, the transaction commitments of the different professional physicians in the specialized hospital were not significant. In Chinese public hospitals, the professional title level is closely related to the income of doctors. The higher the professional title level, the higher the income of doctors. Specialization has a significant impact on the financial and operating performance of hospitals. The research of Vera (2018) et al. shows that the overall financial income of specialized hospitals is higher than that of general hospitals at the same level [28]. Therefore, in specialized hospitals, the income gap between doctors with different professional titles is narrowed. The management of the hospital should comprehensively consider the overall hospital performance and fiscal revenue, and pay attention to improving the affect degree of the professional title level in fulfilling the transaction commitment, which has achieved the purpose of improving doctors’ behavior.

This research also found that, whether in general or specialized hospitals, entry style and whether to hold an administrative position are two main factors influencing physicians’ perceived organizational commitment, and doctors recruited by other institutions who did not hold administrative positions felt lower hospital commitment. The reason may be that doctors transferred by other institutions, compared with previous hospitals, have higher expectations of current hospital commitment. Seruya et al. (2010)also have demonstrated that different entry styles significantly influenced doctors perceived organizational commitment [15]. Doctors in administrative positions are involved in management work in the hospital, so their role in the hospital is different from that of the general doctor. Therefore, they have a better understanding of the hospital management system and personnel system, and they are easier to think from the perspective of the hospital. Consistent with the research of Miedaner et al. (2018), previous cross-sectional research of physicians in German public hospitals shows higher perceived organizational commitment behavior among physicians in administrative positions [26]. However, some other researchers have different results, for example, Abou et al. (2017) showed that whether to hold administrative positions, entry style and academic qualifications did not affect their perception of organizational commitment behavior [16]. After Afsar et al. (2018) investigated the perceptions of Pakistani physicians, they found that whether holding an administrative position was not significant in either univariate or multivariable regression [10]. The reason may be the differences in doctor training plans and career development plans in different countries. Besides, this research found a significant effect of working hours on development commitment scores. Physicians whose working hours are less than 10 years perceived higher development commitments. Most of these doctors are younger, have intermediate or junior titles, and are in their own rapid growth period, so they expect hospitals to provide more on-the-job training and career development opportunities. According to research by Khullar (2018), Chen (2015), and Wei (2021) [32–34], the main reason why young and middle-aged doctors can accept huge work pressure and medium-low pay is that tertiary hospitals can provide the resources needed to improve their ability. Therefore, the hospital management should pay attention to the material and spiritual incentive strategy in the development responsibility. At the same time, they provide a variety of forms of training opportunities for helping one improve self ability and realize self-value, increasing its sense of belonging to the hospital. To some extent, it also can avoid poor hospital development responsibility and hospital youth talent drain [34, 35].

The main contributions of this research are as follows: first of all, this research is different from most research on doctors’ perceived organizational commitment behavior, because it analyzed doctors’ perceived organizational commitment behavior between public general hospitals and public specialized hospitals. The continuous outbreak of COVID-19 has led to a continuous increase in people’s demand for various medical services. China’s large public hospitals are in urgent need of reforms with high quality and refined development [36–38]. Its development model has changed from scale expansion to quality and efficiency improvement, its operation mode changes from extensive management to refined management, and its resource allocation shifts from focusing on material elements to focusing more on human resource development. The research can help healthcare service hospital executives or government policymakers understand the impact of hospital specialization strategies and develop more efficient medical staff incentive systems [39]. Second, this research provides empirical evidence in different contexts, compared with the perceived organizational commitment of physicians in large public hospitals in developed countries such as Germany [25] and Portugal [40]. China is still a developing country with a large population, and its economic development is not advanced. Unlike those political systems in other developing countries such as Iran [41] and Turkey [42], China’s political system has its particularity, and its hospital management system is specialized, too. This research also enriched organizational commitment research in public hospitals in developing countries.

Of course, this research also has some limitations, which could be addressed in future research. First of all, this research uses a sampling method to obtain data. However, there may be biases caused by sampling. The development level and the high-quality resources of the two public specialized hospitals in this research area are at the top of Chinese specialized hospitals. They do not fully represent the situation of other public specialized hospitals in China. Second, this research is simple cross-sectional research that only analyzes the influencing factors of doctors’ perception of hospital organizational commitment in public hospitals and does not consider the effect of government/social corporate responsibility on the results. Third, this is country-specific research, not cross-country research. The findings would provide more valuable information if regional-level factors, as well as country-to-country comparisons, were included in the research. Therefore, it is necessary to include these factors in future research.

## Conclusion

This research used a cross-sectional design to sample four large public hospitals in Beijing and systematically evaluate the factors influencing organizational commitment in different types of hospitals. In this research, organizational commitments varied between different types of public hospitals. The total organizational commitments in specialized hospitals are higher than in general hospitals, especially for transaction commitments and conceptual commitments. There are commonalities and differences in the factors affecting the organizational commitment between general hospitals and specialized hospitals. Mastering these characteristics is especially important in the process of public hospital reform. These hospitals often face demands for overall improvement, while simultaneously maintaining their development strengths. For example, public hospitals in China are in the background of high-quality and refined reform. In future development, general hospitals are required to establish their characteristics and develop their key disciplines. Specialized hospitals should integrate themselves to provide more comprehensive medical services, and the corresponding personnel management measures should also be adjusted differently. Therefore, in the context of Chinese public hospital reform, policymakers and hospital managers should establish more specific and refined employee incentive measures in the future, to meet the expectations of doctors with different hospital types.

### Electronic supplementary material

Below is the link to the electronic supplementary material.

Supplementary Material 1

## Data Availability

The original data supporting the conclusions of this article will be provided by the authors, without being retained. If readers want data from this study, you should contact the corresponding author Junli Zhu.

## Declarations

### The publisher’s instructions

All statements expressed herein only represent those of the authors, and are not necessarily representing those of their affiliated organizations or publishers, editors, and reviewers. Any product that may be evaluated in this article or any statement that may be made by its manufacturer is not guaranteed or approved by its publisher.
