# Supplementary material for: Is there any difference in organizational commitment between general hospitals and specialized hospitals? Empirical evidence from public hospitals in Beijing, China
Source: BMC Health Serv Res. 2023 Dec 12;23:1397. doi: 10.1186/s12913-023-10362-5 (PMC10717447; doi:10.1186/s12913-023-10362-5)
Supplement: Supplementary file 1 — Supplementary Material 1 [file 12913_2023_10362_MOESM1_ESM.docx]

**Questionnaire on the degree of psychological association and daily feelings between doctors and working hospitals**

**Dear Madam / Sir,**

shalom! First of all, thank you very much for taking time out of your busy schedule to participate in this questionnaire survey. This is a questionnaire survey about the psychological correlation of you and the degree of daily feelings. I sincerely ask you to answer the following questions carefully.

Questionnaire filling instructions:

1. For each of the following statements, according to your own opinion, to what extent the statement is in line with your actual situation.

2. The whole research process takes about 10-15 minutes, and there is no right or wrong answer.

3. There is no name in this questionnaire, and the data obtained will only be used for the writing of the paper, without involving commercial use or personal privacy. Here we sincerely thank you for your support and cooperation, I wish you a smooth work, good health!

**The following is personal basic information, including Individual social characteristics , Individual occupational characteristics.**

**Individual social characteristics**

Q1.Your age (one one year old):

A.25- 35 years old B.36-45 years old C.46 - 55 years old

Q2.Your gender：

A.man B.woman

Q3.Your marital status:

A.married B.unmarried

Q4.Your highest degree（Current degree）：

A.doctor B.Master C.bachelor

Q5.Do you have overseas study experience?(More than one year)：

A.Yes B.No

**Individual occupational characteristics**

Q1.Your department category：

A.key disciplinary fields B.Non-key disciplines

Q2.Your working time is _____ hour(s)

Q3.Your title：

A.Associate Chief physician or above B.Physician C.Physician and below

Q4.Whether you hold an administrative position：

A.Yes B.No

Q5.How do you enter the hospital：

A.Campus recruitment B.Transfer of other institutions

**The following sentences describe the current responsibility that the hospital assumes to you. Please type "√" on the number of the corresponding situation according to the conformity degree of your personal subjective perception.**

|  |  | The degree of commitment to this hospital responsibility | | | | |
| --- | --- | --- | --- | --- | --- | --- |
| Research dimension | Related statements | Never promised | Implicit commitment | Strong hint | Definite undertaking | Very clear |
| Concept organization commitment | Q1.The work provided by the hospital is challenging | 1 | 2 | 3 | 4 | 5 |
|  | Q2.The hospital gives you the autonomy in your work | 1 | 2 | 3 | 4 | 5 |
|  | Q3.Hospitals will take their doctors' opinions into full account when making major decisions | 1 | 2 | 3 | 4 | 5 |
| Development Organization Commitment | Q4.The hospital allows you to use your skills and expertise | 1 | 2 | 3 | 4 | 5 |
|  | Q5.The hospital provides you with opportunities for professional learning and technical training | 1 | 2 | 3 | 4 | 5 |
|  | Q6.The hospital provides you with career promotion space and development opportunities | 1 | 2 | 3 | 4 | 5 |
| Trading organization commitment | Q7.The hospital will pay your salary and bonus according to your work performance | 1 | 2 | 3 | 4 | 5 |
|  | Q8.The hospital provides you with stable job security | 1 | 2 | 3 | 4 | 5 |
|  | Q9.The hospital provides you with a fair and reasonable treatment | 1 | 2 | 3 | 4 | 5 |
|  | Q10.The hospital provides you with superior benefits (such as insurance and vacation) | 1 | 2 | 3 | 4 | 5 |
|  | Q11.The hospital provides you with the conditions and resources needed to carry out your work | 1 | 2 | 3 | 4 | 5 |
| Relationship Organization Commitment | Q12.The relationship between the superior and lower levels in the hospital is harmonious and friendly | 1 | 2 | 3 | 4 | 5 |
|  | Q13.The hospital maintains a harmonious relationship between colleagues | 1 | 2 | 3 | 4 | 5 |
|  | Q14.The hospital has great respect for its doctors | 1 | 2 | 3 | 4 | 5 |
|  | Q15.The hospital provided a collaborative working atmosphere | 1 | 2 | 3 | 4 | 5 |
|  | Q16.Hospitals care for your personal development and personal life conditions | 1 | 2 | 3 | 4 | 5 |
|  | Q17. The hospital recognizes your contribution and performance | 1 | 2 | 3 | 4 | 5 |
